# Improvement in Glycemic and Lipid Profiles in Type 2 Diabetics with a 90-Day Ketogenic Diet

**DOI:** 10.1155/2019/8681959

**Published:** 2019-08-14

**Authors:** Chase M. Walton, Katelyn Perry, Richard H. Hart, Steven L. Berry, Benjamin T. Bikman

**Affiliations:** ^1^Metabolism Research Lab, Department of Physiology and Developmental Biology, Brigham Young University, Provo UT, USA; ^2^Insulin IQ, Orem UT, USA; ^3^Revere Health, Orem UT, USA

## Abstract

Because low-carbohydrate diets are effective strategies to improve insulin resistance, the hallmark of type 2 diabetes, the purpose of reporting these clinical cases was to reveal the meaningful changes observed in 90 days of low-carbohydrate (LC) ketogenic dietary intervention in female type 2 diabetics aged 18-45. Eleven women (BMI 36.3 kg/m^2^) who were recently diagnosed with type 2 diabetes based on HbA1c over 6.5% (8.9%) volunteered to participate in an intensive dietary intervention to limit dietary carbohydrates to under 30 grams daily for 90 days. The main outcome was to determine the degree of change in HbA1c, while secondary outcomes included body weight, blood pressure, and blood lipids. The volunteers lost significant weight (85.7 ± 3.2 kg to 76.7 ± 2.8 kg) and lowered systolic (134.0 ± 1.6 to 123.3 ± 1.1 mmHg) and diastolic (89.9 ± 1.3 to 82.6 ± 1.0 mmHg) blood pressure. HbA1c dropped to 5.6%. Most blood lipids were significantly altered, including HDL cholesterol (43.1 ± 4.4 to 52.3 ± 3.3 mg/dl), triglycerides (177.0 ± 19.8 to 92.1 ± 8.7 mg/dl), and the TG : HDL ratio (4.7 ± 0.8 to 1.9 ± 0.2). LDL cholesterol was not significantly different. AST and ALT, plasma markers of liver health, were reported for eight patients and revealed no significant changes. These findings indicate that a short-term intervention emphasizing protein and fat at the expense of dietary carbohydrate functionally reversed the diabetes diagnosis, as defined by HbA1c. Furthermore, the intervention lowered body weight and blood pressure, while eliciting favorable changes in blood lipids.

## 1. Introduction

Insulin resistance is the most common health disorder worldwide [1], affecting half of all adults in countries throughout North America [2], Asia [3], and the Middle East [4]. In addition to its startling prevalence, insulin resistance increases the risk of numerous chronic disorders, such as heart disease [5], dementia [6], and cancer [7]. With such a perspective in mind, the urgency of stemming the global increase in insulin resistance and revealing strategies to reverse it is evident.

Because of the prevalence and pathologies arising from insulin resistance, numerous pharmacological therapies are commonly utilized to improve insulin sensitivity. Several of these therapies seek to either inhibit intestinal glucose absorption (e.g., *α*-glucosidase inhibitors) or increase renal glucose excretion (e.g., SLGT2 inhibitors). However, while varyingly effective in reducing blood glucose and insulin and improving insulin resistance, side effects such as osmotic diarrhea [8] and urinary tract infections [9] curb enthusiasm and widespread use. In contrast to pharmacological attempts to mitigate glucose absorption or accelerate glucose excretion, a rational paradigm is to simply reduce glucose consumption.

As an alternative or complement to conventional drug interventions, lifestyle therapy is a known and proven method of improving insulin resistance [10]. By scrutinizing dietary carbohydrate consumption, which increases blood glucose and insulin, an effective dietary strategy can liberally focus on dietary protein and fat, which have a diminished effect, if any, on blood glucose and insulin [11]. The purpose of this pilot study was to determine whether a 90-day low-carbohydrate dietary change is sufficient to improve markers of insulin resistance and type 2 diabetes.

## 2. Materials and Methods

### 2.1. Subjects

Subjects were recruited from the Provo/Orem area in Utah County. Eligibility was ensured by phone. Retrospective chart review was approved by the Institutional Review Board at Brigham Young University. Inclusion criteria were age 18-45 years with a recent diagnosis of type 2 diabetes mellitus based on HbA1c of 6.5% or higher, along with any features of the metabolic syndrome, including hypertension and dyslipidemia. Exclusion criteria included medication use, previous disease diagnoses, pregnancy, and nursing. Baseline blood tests were performed at the screening visit. Subjects received no monetary compensation.

### 2.2. Intervention

Subjects received an individual visit to provide educational material with weekly visits for the remaining 90 days. Weekly visits consisted of discussions surrounding dietary adherence and ketone measurements. Throughout the study, adherence to the intervention was confirmed with weekly tests to ensure plasma ketones (Precision Xtra, Abbott, Chicago IL) were at or above 0.5 mmol/l (Table 1; average at week 1: 0.9 ± 0.08; average at week 12: 1.3 ± 0.15). Educational material, such as a list of foods/beverages to avoid or consume, and visits were intended to inform and focus on the rationale and implementation of a low-carbohydrate, high-fat diet, with a particular emphasis on the value of adhering to a diet that maintains insulin at low levels (i.e., ketogenic diet). Subjects were encouraged to adhere to a simple rule of “control carbohydrates” (~5% calories), “prioritize protein” (~20-25% calories), and “fill with fat” (~70-75% calories). The key aspect of the dietary intervention was to control carbohydrates by consuming no more than 30 grams per day, coming mostly from nontuberous vegetables and berries. Subjects were encouraged to freely consume protein and fat, including meats, eggs, and cheese; a low-carbohydrate, high-fat shake (Best Fats, Orem UT); or similar. Throughout the study, subjects were encouraged to continue preexisting physical activities.

### 2.3. Outcomes

Key variables included body weight, blood pressure, and blood laboratory tests following a 12-hour fast. Ketone levels were assessed weekly, while all other outcomes were assessed at the beginning and end of the study period. All assessments were obtained by a trained clinician, and all blood analyses were performed by the same lab.

### 2.4. Statistics

Subject data were analyzed via a two-tailed paired *t*-test (GraphPad Prism, San Diego CA) with significance set at a *p* value of ≤0.05.

## 3. Results

### 3.1. Body Weight and BMI

**Figure 1:**
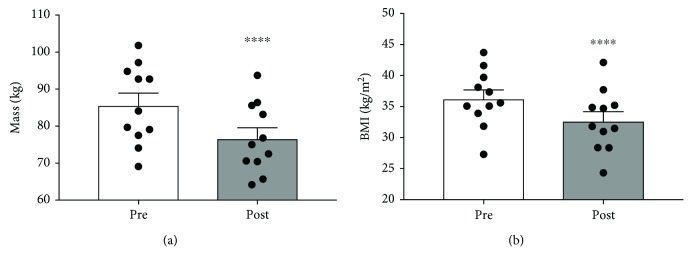
The effect of a 90-day low-carbohydrate diet on body weight (a) and BMI (b) in women (*n* = 11; ^∗∗^*p* < 0.0001).

The subjects were female, aged 38.3 ± 2.6 years, Caucasian, with a mean weight of 85.7 ± 3.2 kg and BMI of 36.3 ± 1.4 kg/m^2^. All eleven subjects lost significant weight, with an average postintervention weight of 76.7 ± 3.2 kg (*p* < 0.0001), further reflected in a reduction in BMI to 32.7 ± 1.5 kg/m^2^ (*p* < 0.0001; Figures 1(a) and 1(b)).

### 3.2. Blood Pressure

**Figure 2:**
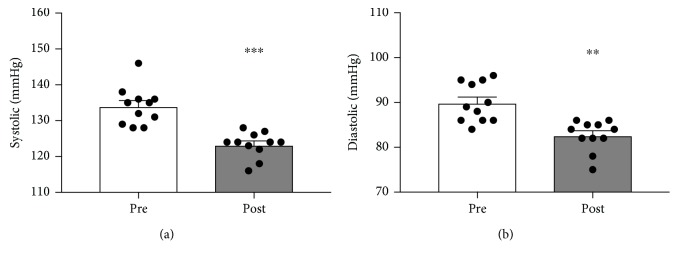
The effect of a 90-day low-carbohydrate diet on systolic (a) and diastolic (b) blood pressures in women (*n* = 11; ^∗∗^*p* < 0.005 and ^∗∗∗^*p* < 0.0005).

Similar to body mass changes, systolic and diastolic pressures dropped substantially. Initial systolic and diastolic blood pressures of 134.0 ± 1.6 and 89.9 ± 1.3 mmHg, respectively, were reduced to 123.3 ± 1.1 (*p* < 0.0001) and 82.6 ± 1.0 mmHg (*p* < 0.005; Figures 2(a) and 2(b)).

### 3.3. Glycated Hemoglobin

**Figure 3:**
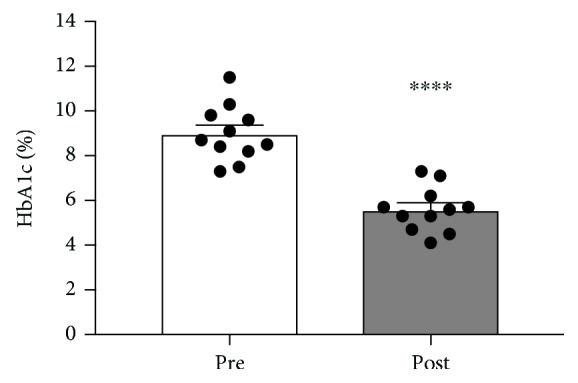
The effect of a 90-day low-carbohydrate diet on HbA1c in women (*n* = 11; ^∗∗∗∗^*p* < 0.0001).

Levels of glycated hemoglobin (HbA1c) are relevant indicators of long-term glycemic control and, therefore, indicative of diabetes severity. As glucose levels are consistently elevated, glucose can nonenzymatically bind hemoglobin on erythrocytes. Traditionally, the diabetic range for HbA1c is 6.5%, and diabetic strategies are primarily focused on getting HbA1c close to or below that cut-off. All subjects were diagnosed with diabetes, with an average HbA1c of 8.9 ± 0.4%. Within 90 days of a LCHF intervention, HbA1c dropped to 5.6 ± 0.3% (*p* < 0.0001; Figure 3).

### 3.4. Blood Lipids

**Figure 4:**
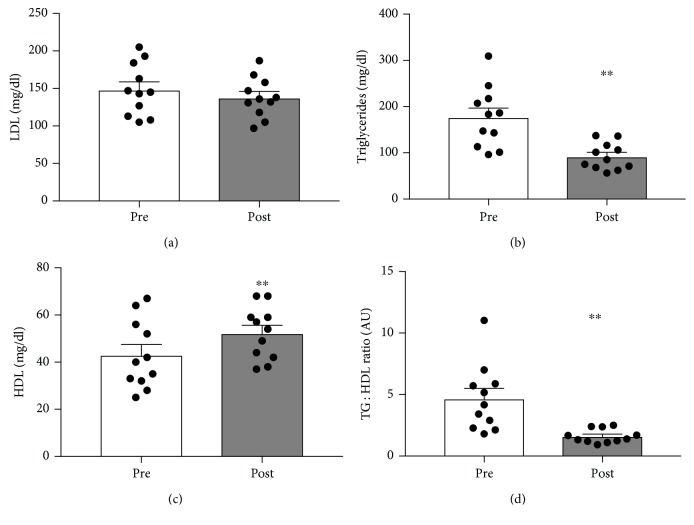
The effect of a 90-day low-carbohydrate diet on LDL cholesterol (a), triglycerides (b), HDL cholesterol (c), and TG : HDL ratio (d) in women (*n* = 11; ^∗^*p* < 0.05).

Blood tests often measure the prototypical blood lipids of interest, which include LDL cholesterol, HDL cholesterol, and triglycerides. Each of these has been classically considered to alter the risk of heart disease to varying degrees. Insofar as heart disease is common among type 2 diabetes [12], the connection between glycemic status and blood lipids is understandable. Similar to HbA1c, the effect of the dietary intervention on blood lipids was substantial. While the reduction in LDL cholesterol (Figure 4(a)) was not significant, triglycerides (TG: Figure 4(b)) dropped significantly (*p* < 0.005), while HDL cholesterol increased (Figure 4(c); *p* < 0.005). The TG : HDL ratio decreased significantly from 4.6 ± 0.8 to 1.6 ± 0.2 (Figure 4(d); *p* < 0.005). Importantly, the TG : HDL ratio is a telling predictor of heart disease, with a much stronger correlation to heart disease than LDL cholesterol [13].

### 3.5. Liver Enzymes

**Figure 5:**
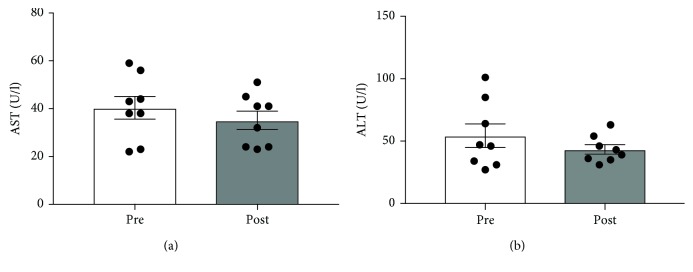
The effect of a 90-day low-carbohydrate diet on plasma AST and ALT in women (*n* = 8).

Various liver disorders are associated with insulin resistance and type 2 diabetes; as such, plasma levels of AST and ALT, considered a reflection of liver health, were determined in eight of the patients (Figures 5(a) and 5(b)). Despite a trend towards a reduction over the 90 days, no significant differences were noted.

## 4. Discussion

The purpose of this manuscript is to report the efficacy of a short-term low-carbohydrate (LC) dietary intervention to improve glycemic control in a group of recently diagnosed subjects with type 2 diabetes. Most importantly, we found that a low-carbohydrate ketogenic diet, wherein carbohydrates constituted roughly 5% of calories, elicited a remarkable reduction in HbA1c from a diabetic level (8.9%) to the standard (5.6%) in only 90 days. Furthermore, the intervention resulted in significant improvements across other cardiometabolic markers, including body weight, BMI, blood pressure, and the triglyceride (TG) : HDL ratio.

Meaningful limitations exist in this study that temper overreaching with regard to the findings. Firstly, the sample size of the report is very small. Secondly, the intervention involved self-selecting patients in a clinical setting, where the subjects were self-motivated and informed of the intervention at the diagnosis of type 2 diabetes. These two concerns are somewhat mitigated when considered in light of evidence from longer and much larger trials, wherein carbohydrate restriction yields similar, albeit less dramatic, outcomes. Very recently, Hallberg et al. [14] found relatively substantial reductions in HbA1c, TG, and other lipids in a nonrandomized study population over one year. Similar findings were observed by Westman et al. [15] after only 24 weeks, with this study benefitting from subject randomization. Again, the carbohydrate-restricted group experienced a significantly greater drop in HbA1c (-1.5% vs. 0.5%) compared with the higher-carbohydrate group. Critically, what these studies have in common with our current report is the level of attention the subjects receive, whether online or in person. This attention may be necessary for the successful outcomes reported, insofar as a LC intervention that receives little instruction appears to carry no benefit in HbA1c when compared with a low-fat group of equally indifferent instruction, despite significantly greater weight loss [16]. Nevertheless, the use of carbohydrate restriction in the treatment of diabetes is now sufficiently well established and accepted that the American Diabetes Association, in their recently published *Standards of Medical Care in Diabetes*, stated, “research indicates that low-carbohydrate eating plans may result in improved glycemia and have the potential to reduce antihyperglycemic medications for individuals with type 2 diabetes” [17].

Despite our efforts to scrutinize insulin resistance, the lack of plasma insulin levels in this study prevents any definitive conclusions with regard to insulin resistance. Future studies from our group will rectify this deficiency in order to more thoroughly explore the effects of our LC intervention on insulin sensitivity. Nevertheless, the lipid changes observed in the subjects in this pilot study reflect an insulin-sensitizing effect, insofar as the TG : HDL ratio, which is considered a surrogate for insulin resistance [18, 19], was significantly reduced.

Because of the relevance of HbA1c in disease outcomes related to diabetes, such as neuropathy [20], nephropathy [21], and especially cardiovascular disease [22], numerous conventional clinical interventions seek to lower glucose and HbA1c by increasing insulin via either insulin therapy or insulin secretagogues, such as sulfonylureas. However, despite the focus on HbA1c and glycemic control, intensive efforts to lower glucose and HbA1c by artificially elevating insulin above the existing hyperinsulinemia common to type 2 diabetes result in an increase in mortality [23]. Herein lies an important aspect of a LC diet as a viable clinical intervention to control glycemia—the ability to reduce HbA1c without exacerbating hyperinsulinemia. To this end, a carbohydrate-restricted approach is intuitive and effective.

## 5. Conclusions

We submit these data as modest, yet undeniable, evidence that a dietary intervention that restricts carbohydrate and emphasizes unrestricted consumption of protein and fat elicits a favorable metabolic state, including the dramatic reduction in HbA1c. Moreover, such an intervention, unlike various insulin secretagogues [24, 25], has the favorable side effect of meaningful body weight and blood pressure reductions.

## Figures and Tables

**Table 1:** Weekly ketone measurements through a 90-day low-carbohydrate diet in women (*n* = 11).

Week	Patient										
	1	2	3	4	5	6	7	8	9	10	11
1	0.7	2.1	0.5	0.3	1.1	1.6	0.6	0.9	1.1	1.2	2.2
2	0.9	1.7	0.7	0.7	1.6	1.5	0.6	0.9	1.4	1.1	1.9
3	1.3	1.7	0.7	0.8	1.4	1.1	0.5	0.7	1.4	1.9	1.4
4	1.0	1.9	0.8	0.7	0.8	1.3	0.7	0.6	1.3	0.8	0.7
5	0.7	1.5	0.5	1.1	1.3	1.9	0.8	0.8	0.9	1.4	0.7
6	0.8	1.3	0.6	1.5	0.9	1.3	0.9	0.9	0.7	1.2	0.6
7	0.6	1.0	0.8	1.2	1.1	1.1	0.9	1.1	0.9	0.9	0.9
8	0.6	0.9	1.1	1.4	1.0	1.0	0.8	1.0	1.3	0.8	1.4
9	0.9	1.3	1.5	1.1	0.8	1.3	0.8	1.1	1.1	0.8	1.4
10	1.5	1.2	0.9	1.7	1.2	1.5	0.8	0.9	1.1	0.6	1.2
11	1.1	0.7	0.8	0.9	0.8	1.1	0.9	0.9	0.9	0.5	1.5
12	0.8	0.9	0.8	1.3	1.1	0.8	1.0	0.7	0.8	0.6	1.3

## Data Availability

The datasets used and/or analyzed during the current study are available from the corresponding author on reasonable request in Graphpad Prism.
